# Sexual conflict in action: An antagonistic relationship between maternal and paternal sex allocation in the tammar wallaby, *Notamacropus eugenii*

**DOI:** 10.1002/ece3.4813

**Published:** 2019-04-05

**Authors:** Amy M. Edwards, Elissa Z. Cameron, Janine E. Deakin, Tariq Ezaz, Jorge C. Pereira, Malcolm A. Ferguson‐Smith, Kylie A. Robert

**Affiliations:** ^1^ Department of Ecology, Environment and Evolution, School of Life Sciences La Trobe University Melbourne Victoria Australia; ^2^ School of Biological Sciences University of Canterbury Christchurch New Zealand; ^3^ Institute for Applied Ecology University of Canberra Canberra Australian Capital Territory Australia; ^4^ Department of Veterinary Medicine University of Cambridge Cambridge UK; ^5^ Cytocell Ltd. Cambridge UK

**Keywords:** *Macropus eugenii*, maternal, offspring sex ratio, paternal, sperm sex ratio

## Abstract

Sex ratio biases are often inconsistent, both among and within species and populations. While some of these inconsistencies may be due to experimental design, much of the variation remains inexplicable. Recent research suggests that an exclusive focus on mothers may account for some of the inconsistency, with an increasing number of studies showing variation in sperm sex ratios and seminal fluids. Using fluorescent in‐situ hybridization, we show a significant population‐level Y‐chromosome bias in the spermatozoa of wild tammar wallabies, but with significant intraindividual variation between males. We also show a population‐level birth sex ratio trend in the same direction toward male offspring, but a weaning sex ratio that is significantly female‐biased, indicating that males are disproportionately lost during lactation. We hypothesize that sexual conflict between parents may cause mothers to adjust offspring sex ratios after birth, through abandonment of male pouch young and reactivation of diapaused embryos. Further research is required in a captive, controlled setting to understand what is driving and mechanistically controlling sperm sex ratio and offspring sex ratio biases and to understand the sexually antagonistic relationship between mothers and fathers over offspring sex. These results extend beyond sex allocation, as they question studies of population processes that assume equal input of sex chromosomes from fathers, and will also assist with future reproduction studies for management and conservation of marsupials.

## 1 INTRODUCTION

Extraordinary sex ratios, those do not adhere to Fisher's homeostatic principle (Fisher, 1930), can contribute to population oscillations, including extreme population crashes (e.g., Grayson et al., 2014; Le Galliard, Ftize, Ferriere, & Clobert, 2005; Rankin, Dieckmann, & Kokko, 2011). For decades, evolutionary biologists have struggled to understand the origins and advantages of offspring sex ratio (offspring SR) biases, including why biases are so inconsistent (e.g., Clark, 1978; Grant, 2007; Hamilton, 1967; Trivers & Willard, 1973). Sex allocation research aims to understand why species experience extraordinary sex ratios (Hamilton, 1967) rather than maintaining homeostatic patterns. However, Fisher's principle applies to population‐level sex ratio biases and to genetic selection for a gene favoring one sex over the other, so the solution to extraordinary sex ratios may be condition‐specific responses. Theory predicts that individual parents would be advantaged if they could adjust offspring SR when fitness returns vary in relation to the parents’ current local conditions (Trivers & Willard, 1973). In mammals, research has focused almost entirely on mothers, partly due to the assumption that the male contribution is under meiotic control and partly due to the differential cost of reproduction with mothers investing more in their offspring than fathers. However, studies investigating the possibility of paternal adaptive sex allocation are increasing and may explain some of the reported inconsistencies in offspring SR (e.g., Edwards & Cameron, 2017; Malo et al., 2017; Vanthournout et al., 2018).

Changes in sperm sex ratios (sperm SR) are likely to influence offspring SR but seminal fluids likely also play a vital role through cryptic male choice (Edwards & Cameron, 2014). Technological advances allow for a fast, cheap, and accurate analysis of sperm SR, which have now been recorded across a several taxa (e.g., Edwards & Cameron, 2017; Malo et al., 2017; Vanthournout et al., 2018). The mechanistic drivers of sperm SR biases remain understudied, but coital rate, environmental contaminants, and age are predicted to play a large role (Edwards & Cameron, 2014). It is also unknown whether studies that do not find biases in sperm SR are not published due to the notion of this being a “nonresult,” and therefore, the prevalence of biases in sperm SR in the literature may be largely artificially inflated (as seen in offspring SR studies, Ewen et al., 1993; Festa‐Bianchet, 1996; Hynes et al., 2005; Miller, Eldridge, & Herbert, 2010). Furthermore, many studies in the past have reported only the population‐level sperm SR, or have combined individual samples prior to analysis (Bugno, Jablonska, & Slota, 2008; Martin, Spriggs, & Rademaker, 1995; Pérez‐Crespo, Pintado, & Gutiérrez‐Adán, 2008); these results are generally not as useful since it is the individual variation that drives offspring SR, not the population average.

Determining the extent of paternal sex allocation and testing if it is adaptive is only the first step in this new avenue of sex allocation research; investigating the interactions with maternal sex allocation is also required. When considering the interests of both parents, one can imagine a series of interaction outcomes: “complementary” where both parents interests lie with one sex, “antagonistic” where the parents interests lie in the opposite sex, and “neutral” where neither parent is interested in deviating from the expected 50:50 ratio (Edwards & Cameron, 2014). If complementary, offspring SR biases could be large, but in the antagonistic scenario, a smaller or non‐existent bias would be predicted if the antagonistic interests cancel each other out. Herein lies a potential explanation for populations that do not appear to conform to predictions from sex allocation theory; if paternal sex allocation influences offspring SR and interacts with maternal interests, particularly through an antagonistic interaction, contradictory results may be obtained if only maternal factors are considered. Understanding these interactions has significant ramifications on our current understanding of the constraints to the evolution of sexual selection and sex ratios (Edwards, Cameron, Pereira, & Ferguson‐Smith, 2016).

Here, we investigate the primary sex ratio in the sperm of a macropod marsupial, the tammar wallaby (*Notamacropus eugenii*; see Eldridge & Coulson, 2015; Jackson & Groves, 2015 regarding change of genus from Macropus). Male reproductive biology in marsupials has been relatively understudied compared to their eutherian counterparts, and to date, there remains no comprehensive investigation into the sperm SR of a marsupial. Initial investigations into sperm SR in the tammar wallaby by Perryman (2012) found a significant bias toward X‐chromosome‐bearing spermatozoa (CBS); however small sample size of captive individuals and low numbers of spermatozoa counted, along with employment of single color (Y) fluorescence in situ hybridization (FISH) renders these results contentious. Single color FISH often results in a bias in the nonstained sex, as all spermatozoa which are nullisomic, did not stain due to methodological error, or that are stained in a phase outside visualization are allocated to the nonstained sex, artificially inflating this count.

We also investigated whether biases found in sperm SR are reflected in offspring SR. Maternal sex allocation studies to date in the tammar wallaby have yielded inconsistent results. Both Sunnucks and Taylor (1997) and Wright and Stott (1990) found support for the Trivers–Willard hypothesis, while Schwanz and Robert (2014) and Perryman (2012) found the opposite relationship in support of the Local Resource Competition hypothesis (Clark, 1978), and Johnson (1989), Inns (1980), and Robert, Schwanz, and Mills (2010) found no relationship between body condition/mass and offspring SR. However, offspring sex in mammalian species is determined at conception, and body condition in all of these studies was measured after conception, usually generally partway though lactation, which could be up to one year after conception (Tyndale‐Biscoe & Renfree, 1987). While the species is good model for investigations of TWH (with substantial variance in male reproductive success, (Ewen et al., 1993; Hynes et al., 2005; Miller et al., 2010) pronounced sexual size dimorphism, spatiotemporal clustering of females, high‐density living [in some populations] resulting in competitive animals accessing more resources (Sunnucks & Taylor, 1997), and a monotocous breeding system (Tyndale‐Biscoe & Renfree, 1987)), the contradictory results mean that, to date, there is no consensus on the status of maternal sex allocation in the tammar wallaby. Here, we test the relative maternal and paternal contributions by measuring both the sperm SR using FISH, and the offspring SR, during both gestation and lactation prior to dispersal.

## 2 METHODS

### 2.1 Study site and species

All fieldwork took place on a private farm west of Parndana (35.82437°S, 137.10822°E), on Kangaroo Island, South Australia. The field site consisted of sheep farming paddocks, where native animals, such as Kangaroo Island kangaroo (*Macropus fuliginosus*), brushtail possum (*Trichosurus vulpecula*), and tammar wallaby (*N. eugenii*) feed on cleared, maintained grassland during the evening. The paddocks are surrounded by corridors of Eucalypt forest, with a thick undergrowth, where these native animals spend their days. Adjoining the sheep paddocks to the south is a *Eucalyptus globulus* plantation.

The Kangaroo Island population of tammar wallaby has become the model species for Macropodidae, and often Marsupialia in general (Hickford, Frankenberg, & Renfree, 2009; Hinds, Tyndale‐Biscoe, Oorchot, & Cooper, 1990; Tyndale‐Biscoe & Renfree, 1987). It is estimated that this island population separated from mainland populations during the last Pleistocene ice age, which ended roughly 10,000 years ago (Kennedy, 1992). However, since mainland populations in South Australia are extinct in the wild, it is thought to have been separated from its nearest relative in Western Australia around 50–100,000 years ago (Oliver, King, & Mead, 1979). Contention remains as to whether Kangaroo Island tammar wallabies are a subspecies or a different species to their Western Australian counterparts (Poole, Wood, & Simms, 1991; van Oorschot & Cooper, 1988).

The monotocous tammar wallabies are highly seasonal breeders. Over 80% of all births occur during the main breeding season, in late January (Flint & Renfree, 1982; Renfree & Tyndale‐Biscoe, 1973), with females having a post‐partum estrus approximately 1 hr after giving birth (Renfree et al., 1989; Rudd, Short, Shaw, & Renfree, 1996). Blastocysts remain in diapause for 11 months and are born in the following breeding season, unless the current pouch young are lost or abandoned prior to the winter solstice (mid‐June). Offspring leave the pouch in October/November, and female offspring enter estrus immediately and mate (Williams, Fletcher, & Renfree, 1998); the resulting single blastocyst remains in seasonal diapause and is born in late January (Paris, Taggart, Paris, Temple‐Smith, & Renfree, 2004; Tyndale‐Biscoe & Renfree, 1987) at the same time as all other births.

Our analysis of sperm SR took place at the end of August 2017, when sperm concentration and total sperm number are highest, and while coagulation rate is low (Paris et al., 2004), which allowed us to collect large numbers of spermatozoa without losing substantial portions to coagulation. However, timing of collection was primarily driven by the management protocols of the farmers.

The offspring SR investigation took place the following year in April and June 2018. The offspring consisted of two cohorts: the “older cohort,” those born during the major season January–February 2018 and resulted from stored blastocysts of matings that occurred between January and June 2017, and the “younger cohort,” those born March–June 2018 resulting from mothers who had lost or abandoned their January/February offspring and reactivated the blastocyst stored from the January–February 2018 mating. Therefore, it is only possible that males in this study sired offspring in the older cohort, as those in the younger cohort are from matings that occurred after the death of the focal males. Conclusions drawn from the offspring SR portion of this study are taken at the population level and not intended to be considered at the individual level.

### 2.2 Sperm sex ratios

#### 2.2.1 Sample collection

Thirty male tammar wallaby bodies were collected after a management shoot (destruction permits issued to private landholders). The shoot took place after dark on two consecutive nights and involved nonselective shooting of all tammar wallabies present. We scavenged sperm samples from 10 males the first night and 20 on the second night. Animals were of unknown age; however, two animals were juvenile in body size and did not have enough spermatozoa present to perform analysis and were excluded from this study. Animals were processed in the field, including body weight and linear measurements (e.g., pes), along with removal of the external scrotum. A field laboratory was set up nearby, where dissection and fixation of spermatozoa took place, between 10 p.m. and 6 a.m. on each night.

The scrotum was incised to allow the testis, epididymis, and vas deferens removal. Spermatozoa were collected from the right cauda epididymis by making 5–6 lateral incisions and allowing the spermatozoa to swim out into approximately 5 ml of phosphate‐buffered saline (PBS, pH 7.4) over a period of 5–10 min. The tissue was removed, and the sample was inverted twice to ensure homogenization. Samples were spun to remove PBS and then fixed in 5 ml Carnoy's fixative (3:1 methanol:acetic acid) and added drop by drop to ensure cells did not clump together. Samples were stored in a portable fridge/freezer at −20°C, until returned to the Canberra laboratory for analysis. The semen sample from the left side, along with both testes were fixed and stored for future use.

#### 2.2.2 Two color fluorescence in situ hybridization

This method was adapted from (Greaves et al., 2001). Chromosome‐specific X and Y paint probes were prepared in the Cambridge laboratory from flow‐sorted tammar wallaby chromosomes amplified and labeled using degenerate oligonucleotide‐primed PCR (DOP‐PCR) as described by (Rens, Fu, O'Brien, & Ferguson‐Smith, 2006). X‐chromosome probes were labeled with Cy3 and Y‐chromosome probes with avidin–fluorescein isothiocyanate (FITC).

The prepared sperm sample was diluted further by adding 5× volume of Carnoy's fixative. Fifteen microliters of sample were dropped onto the center of a clean, glass slide, and left to air‐dry overnight. Samples were denatured in 70% formamide at 70°C for 5 min and then placed immediately into 70% ethanol on ice for 5 min. Samples were then dehydrated further in an ethanol series of 90% and 100% for 3 min each, before being stored in a 37°C incubator until ready for hybridization.

One microliter of each labeled probe was mixed with 8 μl of hybridization buffer (55% formamide, 10% dextran in 1 × sodium citrate buffer [SSC]) and denatured at 70°C for 10 min and then placed immediately onto ice. The denatured chromosome probe was then applied to the slide, covered with a coverslip, sealed using rubber cement, and hybridized for 24–48 hr at 37°C in a moist container.

Post‐hybridization washes consisted of six 2‐min baths at 45°C. Three of 50% formamide in 2 × SSC, and three of 2 × SSC. Slides were dehydrated in an ethanol series for 1 min at each step. Slides were counterstained using 4′6‐diamidino‐2‐phenylindole/ml (DAPI) and mounted in an anti‐fade solution (Vectashield; Vector Laboratories, Burlingame, CA, USA).

#### 2.2.3 Sperm counting

Spermatozoa were observed using a Zeiss Axio Scope.A1 (Carl Zeiss AG, Oberkochen, Germany), fitted with Cy3‐, FITC‐, and DAPI‐specific filters. A minimum of 500 spermatozoa per animal (average 685) were counted in order to obtain sperm sex ratios from each male. Images were collected using Leica Q‐FISH software (Leica Microsystems Imaging Solutions, Cambridge, UK) with a CCD camera, through the ×63 oil‐immersion objective.

The slides were quality checked, for accurate staining and minimal clumping of cells. Areas of the slide that did not stain well, or where cells were clumped were not counted. Sperm that did not have a stain were not included in the count, as it was impossible to determine accurately whether this sperm was nullisomic or did not stain due to methodological error. No disomic or multisomic sperm were seen. Accurately stained spermatozoa were counted starting at an arbitrary location in the upper left section of the sample, moving downward and right, until a minimum of 500 spermatozoa per individual were achieved. Multiple slides per animal were analyzed if required.

### 2.3 Offspring sex ratios

#### 2.3.1 Data collection

Offspring SR were measured using two methods; live trapping and release, and collection of bodies after a management shoot (destruction permits issued to private landholders).

Trapping took place during April and June 2018 and consisted of a total of 390 trap nights. Thomas traps were baited with a combination of stock feed and carrots, set prior to sunset and checked routinely throughout the evening. Captured animals were transferred to hessian handling bags and tagged for individual identification in both ears using Monel (size 12) self‐piercing tags (National Band and Tag Co., New York). Body measurements including mass, pes length, and tail circumference were taken. Animals were sexed, and pouches of females were inspected for offspring. Offspring were sexed without removal from the pouch to ensure minimal interference. Animals were released from the handling bag immediately after processing. During the trapping session, a total of two pouch young were observed.

At the end of the June trapping session, a management shoot took place on the farm. The following morning, 21 female wallaby bodies were processed in a similar method as outlined above. However, all offspring were removed from pouch, sexed, and weighed. As a result of the management shoot, a total of 17 pouch young were recorded.

### 2.4 Statistical analysis

Body condition of both males and females was calculated from the residuals of an ordinary least squares linear regression of body mass and pes length (Schulte‐Hostedde, Millar, & Hickling, 2005).

#### 2.4.1 Sperm sex ratio analysis

We used repeated goodness‐of‐fit tests (G‐test) to investigate whether the individual and the population‐level sperm sex ratios differed from the expected 50:50 ratio and also to investigate the heterogeneity within the population. The additive ability of the G‐test allows us to test each sample individually and also to investigate the heterogeneity in the population (a measure of statistical difference between samples in the population) (McDonald, 2014). Further to this, we undertook a general linear model with binomial error to verify the results of the population‐level analysis.

A generalized linear model, with binomial error was run on the full data set to investigate whether body condition, testis weight, and volume influenced the sperm sex ratio of the males, where testis volume was calculated as the volume of a prolate spheroid.

#### 2.4.2 Offspring sex ratio analysis

Offspring measured were from two birthing cohorts; the older cohort (>100 g), born in the major breeding season, January–February 2018, and resulting from stored blastocysts from matings that occurred during 2017; and the younger cohort (<60 g), born March–June 2018, result from stored blastocysts from the January–February 2018 mating period. As the difference between cohorts was visually obvious, the two offspring captured during trapping were placed into these categories based on visual inspection of animal size, as we did not obtain mass from offspring permanently attached to the teat.

A general liner model with binomial error was used to analyze the total offspring sex ratio and the offspring sex ratios of each individual cohort. A generalized linear model, with binomial error was also run on the full data set to investigate whether maternal body condition influenced offspring sex ratios.

## 3 RESULTS

### 3.1 Sperm sex ratio analysis

**Figure 1:**
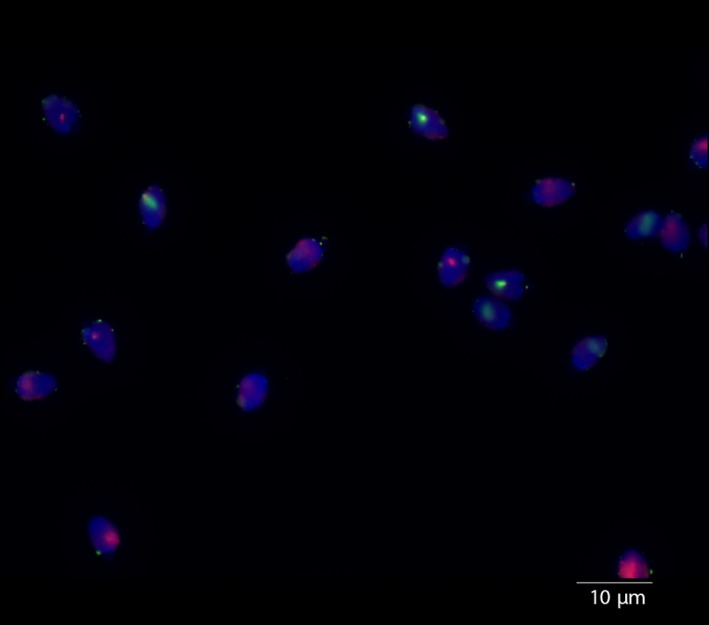
Representative image showing the fluorescence in situ hybridization signals in tammar wallaby (*Notamacropus eugenii*) interphase nuclei. X‐chromosomes labeled with Cy3 and Y‐chromosomes labeled with avidin–fluorescein isothiocyanate (FITC). The two fluorescent domains, red and green indicate the X‐ and Y‐chromosomes, respectively

**Figure 2:**
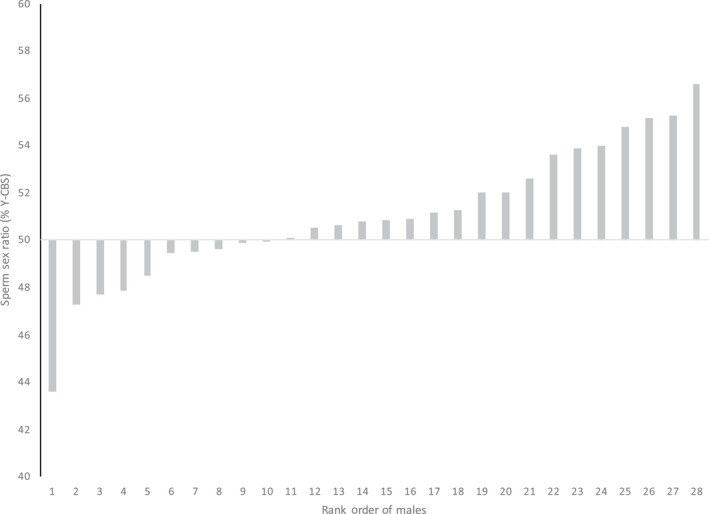
Sperm sex ratios of individual male tammar wallabies (*Notamacropus eugenii*) presented in rank order of lowest to highest % Y‐chromosome‐bearing spermatozoa (CBS)

Representative FISH images of the stained sperm preparations are shown in Figure 1. The population average was 51.05% Y‐CBS, which is significantly different from the expected 50:50 ratio (GLM [CI]: 0.0159, 0.0725). The heterogeneity test indicated that the sperm sex ratios from the different samples were not homogeneous (Figure 2; Heterogeneity G = 54.914, DF = 27, *p* < 0.01).

Within the population, the two most extreme values (minimum and maximum % Y‐CBS) were each significantly different from the expected 50:50 ratio and came from individual 8, whose sperm sex ratio was 43.62% Y‐CBS (G = 9.718, DF = 1, *p* < 0.01), and individual 21 whose sperm sex ratio was 56.58% Y‐CBS (G = 8.845, DF = 1, *p* < 0.01). Further to this, there were six individuals whose sperm sex ratios departed from the expected 50:50 ratio (Figure 2). This is further supported by the significant heterogeneity score from the population. There was no relationship between sperm sex ratio and male body condition (Pr[>Chi] = 0.24), testis weight (Pr[>Chi] = 0.33), or testis volume (Pr[>Chi] = 0.85).

### 3.2 Offspring sex ratio analysis

**Figure 3:**
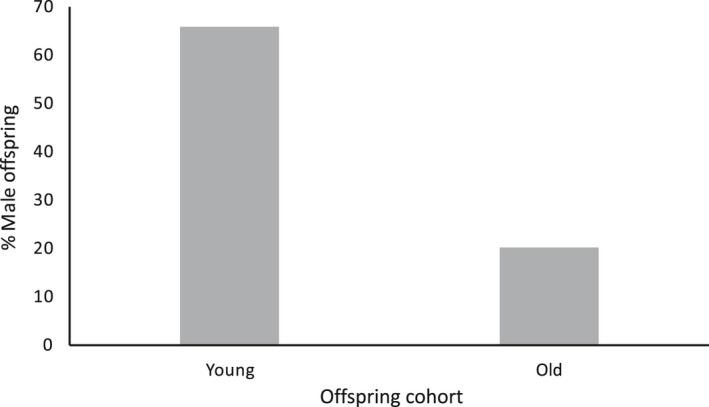
Sex ratio of tammar wallaby (*Notamacropus eugenii*) offspring divided into the two age cohorts based on body size. Younger cohort with mass <60 g, older cohort with mass >100 g

The total offspring (*n* = 19) sex ratio was 42% male, which did not depart significantly from the expected 50:50 ratio (GLM [CI]: −1.267, 0.586). After dividing the offspring into two cohorts, it was noted that the younger cohort (*n* = 9) exhibited a nonsignificant trend toward surplus male offspring (66.6% male; GLM [CI]: −0.639, 2.249), while the older cohort (*n* = 10) exhibited a significant female bias (20% male; GLM [CI]: −3.277, −0.002; power = 0.82; Figure 3). There was no relationship between offspring sex ratio and maternal body condition (Pr[>Chi] = 0.78) or tail circumference (Pr[>Chi] = 0.88).

## 4 DISCUSSION

Here, we have shown a slight, yet statistically significant, population‐level Y‐chromosome bias of spermatozoa of male tammar wallaby, along with significant individual variation, with individuals experiencing significant biases in both directions. In addition, the offspring SR at the time of birth appears to also be male‐biased (although only approaching significance), matching the Y‐chromosome bias, but the weaning cohort is female‐biased. These results show the potential for paternal sex allocation in a marsupial since the sperm sex ratios are variable. These results also indicate a potential antagonistic interaction between fathers and mothers, where sperm SR is biased in the same direction as birth offspring SR but not weaning offspring SR.

Individual and population‐level sperm SR biases are not as rare as previously thought (e.g., Beckett, Martin, & Hoar, 1989; Edwards & Cameron, 2017; Edwards et al., 2016; Saragusty et al., 2012; Vanthournout et al., 2018). Here, we show a population average of 50.05%, which may seem biologically insignificant at first glance; however, when considering sperm counts per ejaculate in this species (Paris et al., 2004), this small deviation results in hundreds of thousands, if not millions, more Y‐CBS than X‐CBS. The general assumption that sperm SR should be 50:50 originates from meiosis creating an equal number of X‐ and Y‐chromosomes. However, a significant amount of time between meiosis and sperm maturation leaves open an opportunity for biases to arise (Cameron, Edwards, & Parsley, 2017). The point at which biases form is yet to be determined, but differential absorption or survival is a likely mechanism. The driving forces behind these changes are often not attributable, but age (Martin et al., 1995; Sartorelli, Mazzucatto, & Pina‐Neto, 2001), environmental contamination (Chandler, Canal, Paul, & Moser, 2002; Chandler et al., 2007; Hilsenrath, Swarup, Bischoff, Buster, & Carson, 1997), and coital rate (Perez‐Crespo, Pintado, & Gutierrez‐Adan, 2008; Scialli et al., 2010; Tiido et al., 2005) are among some of the more common hypothesized explanations.

Due to the free‐living nature of the focal male tammar wallabies in this study, we are unable to attribute biases in sperm sex ratio to any individual factor. Our data suggest that sperm SR are not related to male body condition, or to either testis mass or volume, a proxy for sperm‐producing tissue (Arai, Kitahara, Horiuchi, Sumi, & Yoshida, 1998). Wallabies in this study reside on farming properties and nearby forestry lands and are likely exposed to a number of anthropogenic environmental contaminants as a result of past and current practices. This population is also subjected to haphazard management schedules, whereby population control is not undertaken to the same degree each year; this may also play a role in both maternal and paternal sex allocation. Seasonal variation in sperm SR also potentially exists, as seasonal variation in testes and accessory gland size has been noted in the tammar wallaby (Paris et al., 2004); however, as individual variation in sperm SR is so high, research investigating seasonal changes would need to be performed at the individual level.

The population‐level bias in sperm SR warranted the need to investigate the population‐level OSR. While this study cannot provide information on individuals nor provide causal explanations for biases measured, it is the first study to measure both sperm SR and offspring SR in a wild marsupial. The bias toward Y‐CBS is reflected with a nonsignificant trend toward male offspring in the birth offspring SR. The difference between the 66.6% male offspring in the younger cohort and the 20% male offspring in the older cohort suggests the possibility of maternal adaptive control between birth and weaning. Marsupials have the “pouch advantage” (Robert & Schwanz, 2011), whereby offspring are born at a relatively early stage of development, with females yet to invest large amounts of energy into offspring (Hayssen, Lacy, & Parker, 1985; Renfree, 1983; Tyndale‐Biscoe, 2005). Eutherian mothers would require signals of offspring sex at an early stage to selectively abort after the peri‐conceptual stage (Hardy, 1997); it is, therefore, remarkably easier for a marsupial mother to abort or abandon the current offspring (Robert & Schwanz, 2011). It is possible that the trend toward male offspring in the younger cohort, the suggested “birth offspring SR,” was driven by sperm SR and is not in line with maternal interests, resulting in an antagonistic relationship between parents. If mothers are selectively aborting male offspring then we may have witnessed sexual conflict in action as a result of an antagonistic relationship between sex allocation interests; however, it is also possible that this result is due to environmental conditions and not adaptive control. Central Kangaroo Island suffered a relatively dry summer this year, with December through March experiencing 40% less than the average rainfall (Station: 22837; www.bom.gov.au; accessed 18/07/2018), resulting in a potential lack of resources available to lactating mothers. Male young of many species require more resources than their female counterparts (e.g., Clutton‐Brock, Albon, & Guinness, 1981; Froy, Walling, Pemberton, Clutton‐Brock, & Kruuk, 2016; Gomendio, Clutton‐Brock, Albon, Guinness, & Simpson, 1990); therefore, the results seen here may be a consequence of male abandonment due to lack of resources rather than maternal choice per se. While this may still constitute a form of adaptive sex allocation, in that mothers are unable to raise high‐quality male offspring, the argument as to whether differential loss as a result of cost is true adaptive maternal sex allocation remains to be seen. However, there is evidence to support that male and female offspring of the tammar wallaby may not exhibit sex‐specific investment costs on the mothers during lactation, as growth is independent of sex prior to leaving the pouch (Murphy & Smith 1970) and cross‐fostering offspring by sex imposed limited costs on the mother (Robert et al., 2010; Schwanz & Robert, 2016), and therefore, a maternal adaptive control scenario may be the more feasible explanation. Regardless of the number of uncontrolled variables in this study, the results presented here suggest that interactions between maternal and paternal interests in sex allocation require further consideration.

Female body condition was not related to offspring SR in the present study, and therefore, one could draw the conclusion that this population does not follow the expected traditional relationship between maternal condition and an increase in offspring SR (Trivers & Willard, 1973). However, it should be noted that the underlying mechanisms likely driving this relationship are in action at the point of conception, as this is when offspring sex is determined (Cameron 2004). Body condition of these females was measured at time of capture or death; this potentially being upwards of 12 months after the offspring was conceived (Tyndale‐Biscoe & Renfree, 1987). It is therefore unsurprising that maternal body condition was not related to offspring SR considering the variability in female condition across the season (Schwanz & Robert, 2012). The current study design did not allow for collection of body condition data at the point of conception, and further research would be required to determine whether this population conforms to the relationship predicted by Trivers and Willard (1973).

Implications of results such as these extend beyond the general confines of sex allocation research (Edwards & Cameron, 2016). Many lines of research assume equal contributions of X‐ and Y‐chromosomes from fathers, for example, studies of dispersal using Y‐chromosome microsatellite markers and diversity (e.g., Eriksson et al., 2006; MacDonald, Fitzsimmons, Chamber, Renfree, & Sarre, 2014), along with many studies regarding population processes in ecology. Further understanding in sperm SR research could help improve reproduction for conservation breeding or livestock (e.g., Ideta et al., 2009), by reducing overall wastage and increasing production efficiency. Further, studies in managed species, such as the tammar wallaby, may directly lead to improvements in management processes and a potential reduction in overall effort required by farmers and landholders.

Further research is required to ascertain the generality of and factors contributing to sperm SR biases in marsupials. Potential controlled experiments may isolate drivers such as coital rate, environmental conditions/contaminants, and age. Studies of captive animals could test for a direct relationship between male sperm SR and his subsequent or lifetime offspring SR, gaining further insight into the interaction between parental interests. This study suggests that paternal sex allocation does exist in marsupials and that all future studies of sex allocation would ideally consider paternal, as well as maternal interests. Previous studies that failed to conform to theory or produced interannual differences (Schwanz & Robert, 2014) may have been better explained with consideration of paternal influences.

## CONFLICT OF INTERESTS

We have no competing interests.

## AUTHOR CONTRIBUTIONS

AME designed the study, completed all field and laboratory protocols, analyzed the data, and drafted the manuscript. EZC designed the study and helped draft the manuscript. JED and ET assisted with FISH techniques and helped draft the manuscript. JCP and MAF flow sorted the chromosome probes and helped draft the manuscript. KAR designed the study, assisted with fieldwork and helped draft the manuscript. All authors gave final approval for submission.

## ANIMAL ETHICS

Sperm samples were collected under scientific permit E26595‐1 from Government of South Australia, Department of Environment, Water and Natural Resources. Animal ethics permit AEC18‐08 was obtained for trapping wallabies from La Trobe University Animal Ethics Committee. Offspring sex ratios from trapped and deceased animals were measured under scientific permit M26743‐2 from Government of South Australia, Department of Environment, Water and Natural Resources.

## Data Availability

Data are available on Dryad (https://doi.org/10.5061/dryad.d83h613).
